# Medical Diagnosis with Special Reference to Practical Medicine

**Published:** 1891-01

**Authors:** 


MEDICAL DIAGNOSIS, WITH SPECIAL REFERENCE TO PRACTICAL MEDICINE. A Guide to the Knowledge and Discrimination of Diseases. By J. M. DACOSTA, M. D., LL. D., Professor of Practice of Medicine and Clinical Medicine at the Jefferson Medical College, Philadelphia; Physician to the Pennsylvania Hospital; Consulting Physician to the Children's Hospital, etc., etc. Illustrated with engravings on wood. Seventh edition, revised. 8vo; pp. 995. Philadelphia: J. B. Lippincott Company. London: 10 Henrietta street, Covent Garden. 1890. Price, cloth, $6.00.

The first edition of DaCosta's Medical Diagnosis appeared in 1864, since which time it has been considered a text-book on the subject of which it treats, having been adopted by all the medical colleges worthy the name in this country. Indeed, it may be said that the treatise is as near a classic as any contemporaneous medical literature ever can be, since no physician can afford to be without it, and every teacher *must* possess it.

The progress in medicine in the past twenty-six years has been great, and methods of diagnosis have been ever changing, but "DaCosta," from the first date of its issue, has held its place during all this period as an able and trustworthy exponent of all that was known or worth possessing in the field it seeks to cover.

We cannot undertake to review a book that is so familiar to our readers; it would be next to an insult to their intelligence to expect them to read it if we should; but we could not easily forgive ourselves for not expressing an opinion of the great work our distinguished countryman, the author, has done for his fellow-physicians, and, through them, for humanity, in so industriously pursuing his important field of instruction.

Dr. DaCosta is a graphic writer, and uses English in its purest idiom; his style is direct, expressive, finished, and attractive; but it is never prolix, and seldom fails to convey the author's meaning in the fewest words consistent with clearness. He rarely expresses himself in epigrams, yet his sentences are not involved, and are often rounded in the most agreeable manner.

This seventh edition of this book must necessarily command a large sale, as the additions to the subject are many since the previous one, and no one can afford to read a back number in a department of medicine that follows anatomy and physiology so closely as to fundamental importance.

The publishers have presented the book, as is their wont, in a most attractive dress.

